# Non-surgical Management of a Large Periapical Lesion: A Case Study of the Successful Application of a Modified Triple Antibacterial Paste

**DOI:** 10.7759/cureus.62349

**Published:** 2024-06-14

**Authors:** Srushti Awghad, Joyeeta Mahapatra, Amit Reche, Ankita Burse, Aradhana Kibe

**Affiliations:** 1 Conservative Dentistry and Endodontics, Sharad Pawar Dental College and Hospital, Datta Meghe Institute of Higher Education and Research, Wardha, IND; 2 Public Health Dentistry, Sharad Pawar Dental College and Hospital, Datta Meghe Institute of Higher Education and Research, Wardha, IND; 3 Conservative Dentistry and Endodontics, Bharati Vidyapeeth Dental College and Hospital, Sangli, IND

**Keywords:** non-surgical root canal treatment, double antibiotic paste, modified triple antibiotic paste, triple antibiotic paste, periapical lesion, non-surgical management

## Abstract

Infection of the dental pulp involves mainly Gram-negative, anaerobic bacterial flora and due to this infection, the periapical area experiences an immunological response, which is termed a periapical lesion. This lesion may appear as a radiolucent (dark) area on X-rays, which indicates periapical inflammation and infection. Its prevalence depends on factors such as age, oral health maintenance, and dental care. Men are more likely to be affected by this infection than women. There are two modalities for the treatment of periapical lesions: surgical or non-surgical endodontic therapy. The modified triple antibiotic paste (TAP) comprising ciprofloxacin, metronidazole, and clindamycin in the ratio of 1:1:1 was first prepared expressly to treat the teeth with necrotic pulp and to support the protocol for revitalization and regrowth. The treatment was very successful in eliminating germs from the root canal system. It provides broad-spectrum antimicrobial activity against a wide range of bacteria commonly associated with endodontic infections. The modified TAP is usually inserted into the canal for a predetermined amount of time and then removed followed by the irrigation of root canal, which helps to eliminate the microorganisms from the root canal. The non-surgical treatment should always be the first choice over the surgical approach so as to avoid a more invasive procedure.

## Introduction

A periapical lesion refers to a pathological condition surrounding a tooth root's apex or tip. This condition often involves inflammation or infection of the tissues surrounding the tooth root, including the bone. It can be caused by untreated dental caries, trauma, or other factors leading to bacterial infection [1]. Infection of dental pulp involves mainly Gram-negative, anaerobic bacterial flora and due to this infection, the periapical area experiences an immunological response that is termed a periapical lesion [2]. The primary cause of periapical lesions is root canal infection, whether they are abscesses, granulomas, or cysts [3]. In the context of dentistry, a periapical lesion may appear as a radiolucent (dark) area on X-rays, which indicates periapical inflammation and infection [4]. Radicular cysts, abscesses, and dental granulomas are the three main categories of periapical lesions [5]. A periapical cyst can be identified by the clinical and radiological findings. Radiographically, it is situated at the tip of the non-vital teeth but sometimes can occur at the mesial or distal surface of the root at the opening of the accessory canals [6]. The border of the periapical cyst appears well corticated, but the loss of cortical border is seen if the cyst is infected and the lining of the cyst is either circular or curved unless it gets affected by nearby structures. [7]. There are two modalities for the treatment of periapical lesions: surgical or non-surgical endodontic therapy. The maxillary lesions heal more rapidly than those in the mandible as it has a larger circulatory network than the mandible (maxilla > mandible). The anterior lesions of the jaw heal faster than the posterior ones (anterior region > posterior region) due to the close proximity of the buccal and lingual plates in the anterior segments [8].

Prevalence

The prevalence of periapical lesions depends on factors such as age, oral health maintenance, and dental care [8]. Untreated dental caries and other conditions that cause infection or swelling of the dental pulp may also have a detrimental effect on the prevalence [8]. The relative frequency of occurrence in all intraoral cysts is 52-68% [9]. The more frequently involved parts of the jaw bone are the anterior region of the maxilla and the premolar region of the mandible. The highest prevalence of periapical infection is seen in patients in their third decade of life and men are more likely than women to get affected by it [10].

## Case presentation

**Figure 1:**
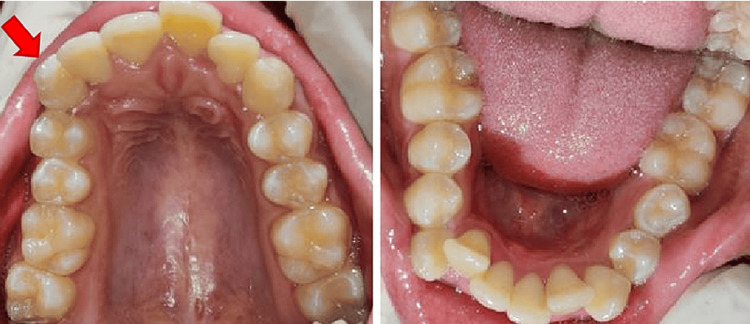
Intraoral clinical photograph of the upper and lower arch. Arrow denotes tooth number 13.

A 23-year-old female patient reported to the tertiary care center in Sawangi (Meghe), Wardha, India, with the chief complaint of pain in the upper front region of the jaw for four days. The patient started experiencing pain in the maxillary front region of the jaw four days back, which had a gradual onset at the beginning, was moderate in intensity, tingling in character, intermittent in nature, and non-radiating. The pain aggravated while eating. The patient had a history of a sports injury, which led to trauma in the front region of the jaw seven years back, resulting in a laceration of her lower lip. The patient was asymptomatic for the next seven years after the accident. She developed pain in the right upper anterior region one year back, which subsided on its own. There was no history of swelling, night pain, or pus discharge. The patient neither had any medical history nor was on any medication. Dental history was insignificant. The face was bilaterally symmetrical. The temporomandibular joint movement was smooth and synchronous with no clicking sound. Lips were competent. No extra-oral swelling was seen. On intraoral clinical examination, all the teeth were present (Figure 1).

**Figure 2:**
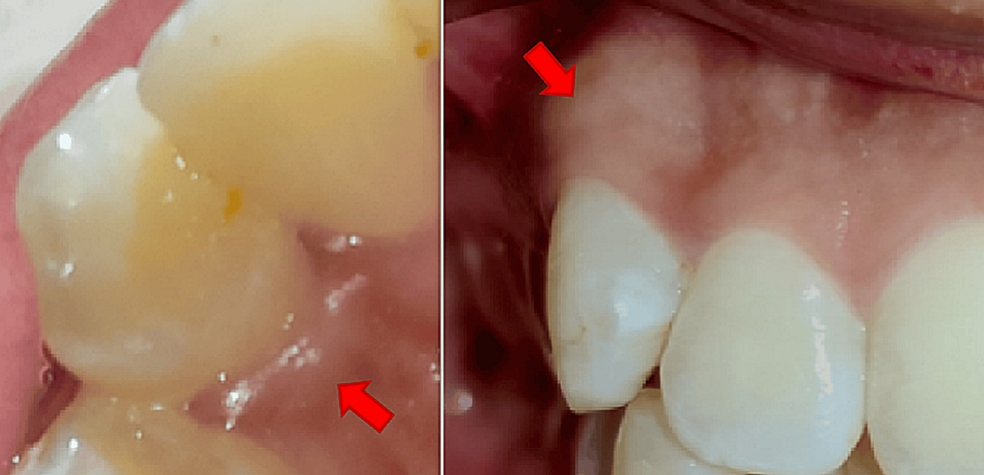
Intraoral clinical photograph of the palatal view and the labial view in occlusion of tooth number 13.

**Figure 3:**
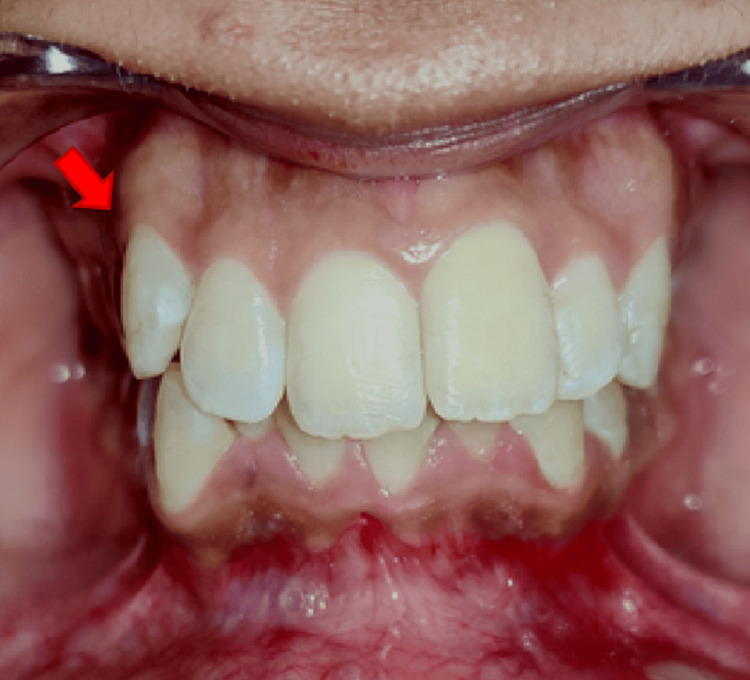
Clinical photograph showing the intraoral profile view of the patient. Arrow denotes tooth number 13.

None of the teeth displayed any carious lesion including the right maxillary canine (i.e., tooth number 13) (Figures 2, 3). However, tooth number 13 was tender on vertical percussion. On palpation, the alveolar region near the root surface of tooth number 13 was tender. Neural sensibility testing (cold test) was done using Endo-Frost (Coltene Vitality Control Endo-Frost, Langenau, Switzerland) on tooth numbers 11, 12, 13, 14, 21, 22, 23, and 24. All the teeth gave a normal response to the cold test except tooth number 13, which gave no response.

**Figure 4:**
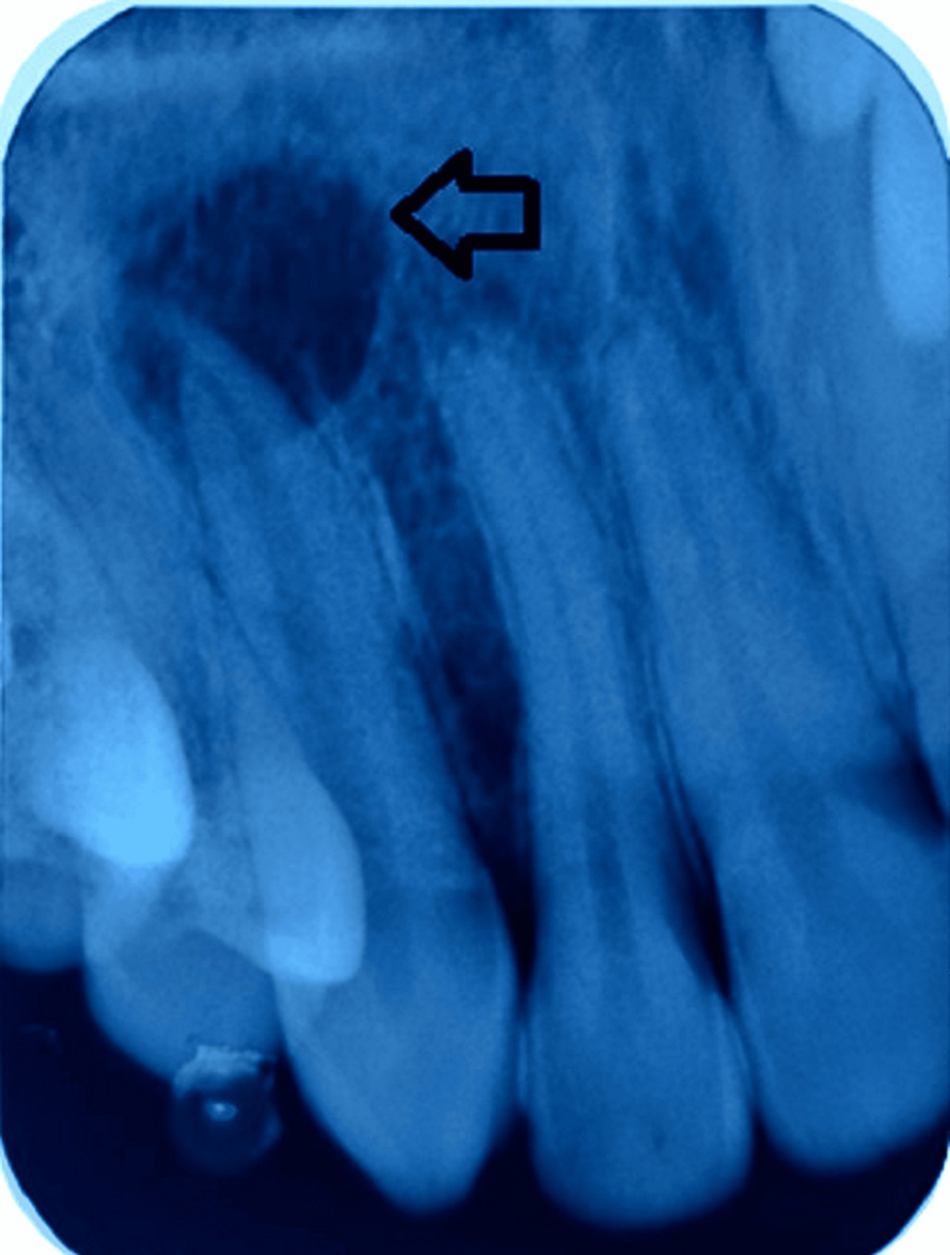
Intraoral periapical radiograph showing periapical radiolucency associated with tooth number 13.

A radiographic examination was undertaken using an intraoral periapical radiograph (IOPA). The coronal structure of tooth number 13 displayed intact enamel and dentin suggestive of no carious involvement. In the radicular portion, a well-defined periapical radiolucency of size 13mm x 15mm was observed to be associated with tooth number 13 (Figure 4).

The final diagnosis was pulp necrosis with apical periodontitis in relation to tooth number 13. A differential diagnosis of periapical abscess, periapical granuloma, or periapical cyst was suggested as per radiographic findings [7]. However, this could not be verified as a histopathological examination was not performed. Hence, a non-surgical root canal therapy was planned as a treatment option for treating tooth number 13. 

**Figure 5:**
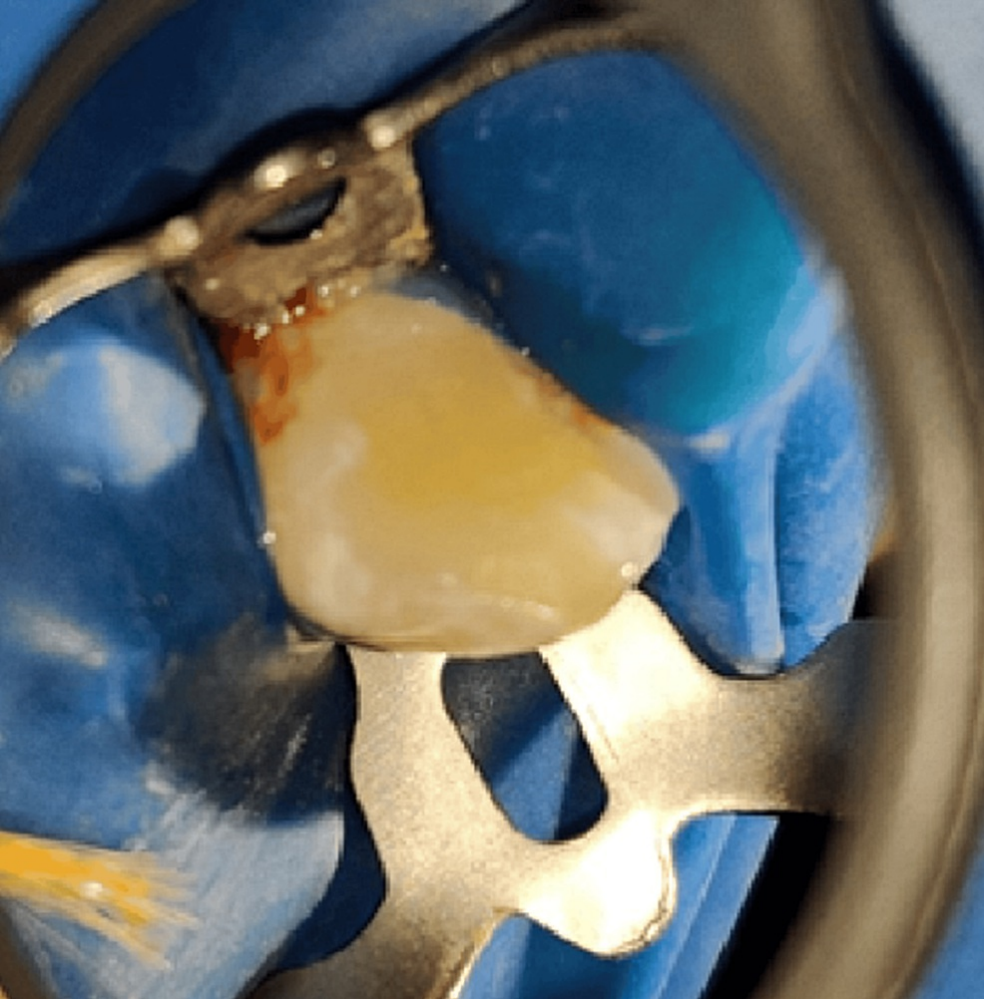
Rubber dam isolation of tooth number 13.

**Figure 6:**
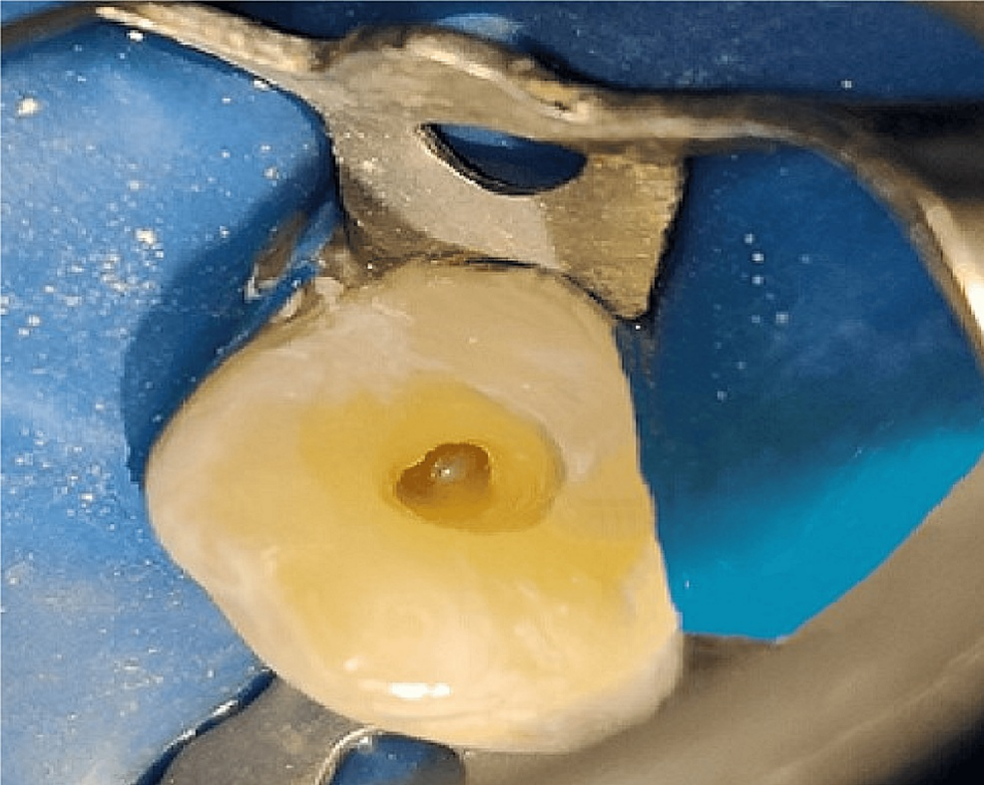
Access opening done with tooth number 13.

Local analgesic was administered with 2% lignocaine hydrochloride along with adrenaline (concentration was 1:2,00,000). Then the rubber dam was used to isolate the tooth (Figure 5). Access cavity was prepared with round bur (BR-45, Mani, Utsunomiya, Japan) and Safe-end (EX-24, Mani, Utsunomiya, Japan) (Figure 6). Patency filing was done up to number 15 K-file with the tooth number 13. A yellowish turbid fluid discharge was observed from the cavity of 13.

**Figure 7:**
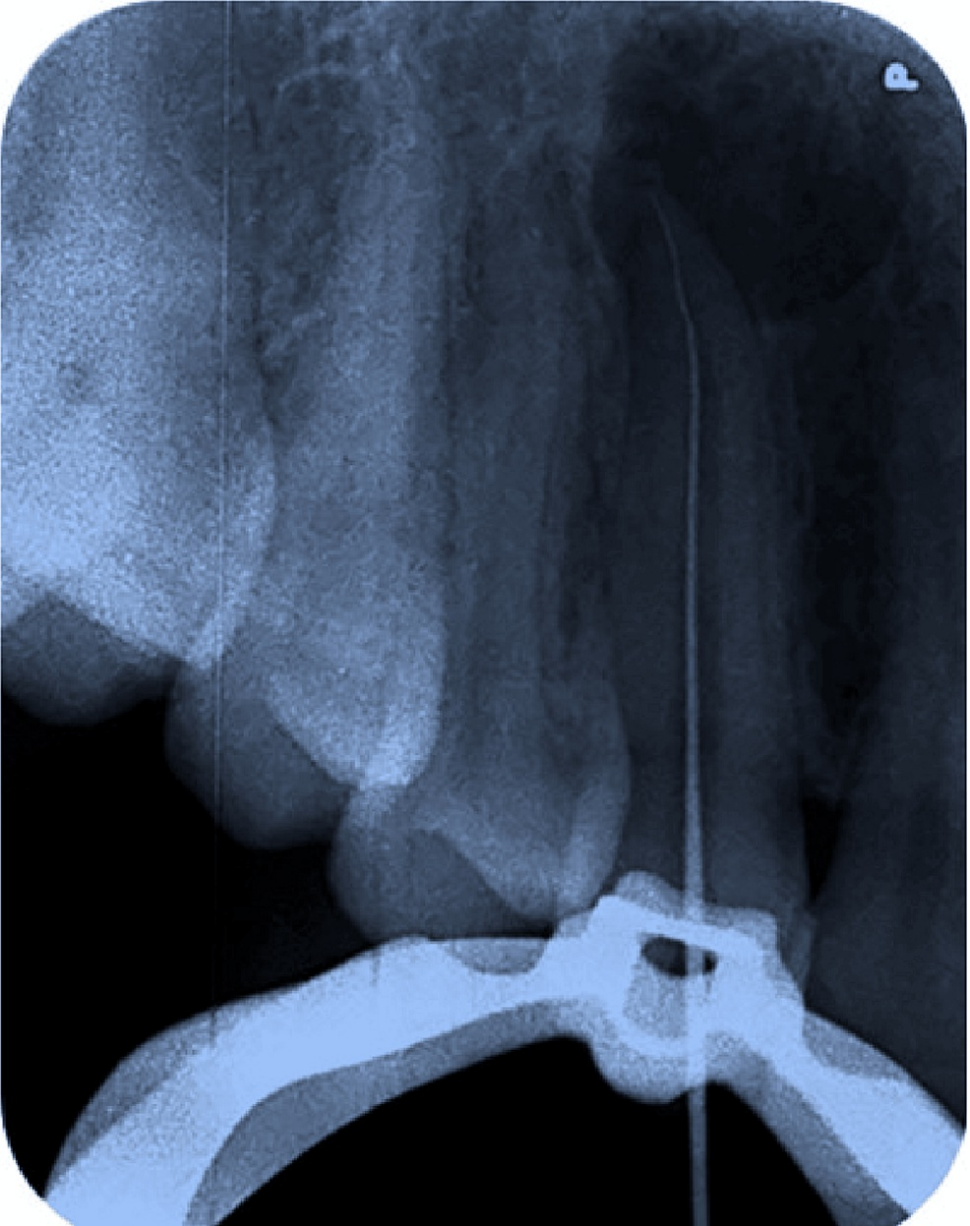
Radiographic working length determination with the help of a number 15 K-file with the tooth number 13.

Working length was determined using an electronic apex locator (Root Zx Mini, J Morita, Tokyo, Japan), which was 27 mm in relation to tooth number 13 (Figure 7). This measurement was verified using a radiograph with the help of a number 15 K-file. For the biomechanical preparation, we required a K-file of 31 mm (no. 15-40 and no. 45-80). The first file to bind was ISO 45 K-file. The apical preparation was enlarged to a file three sizes larger than the first binding file. Thus, the master apical preparation size was up to ISO 60 K-file of 2% taper. A step back of 1 mm each from the working length was terminated up to number 80 K-file of 2% taper. Circumferential filing of the root canal was done using number 35 H-file. The canal was thoroughly irrigated with 3% sodium hypochlorite solution followed by copious irrigation with 0.9% saline for a complete elimination of pulp and debris. The final rinse of the canal was done with 2% chlorhexidine digluconate solution. All the irrigants were activated in the canal using an Endoactivator (Dentsply Sirona, Tulsa, OK, USA).

A modified triple antibiotic paste was prepared, which had a combination of three antibiotics: clindamycin (100mg), ciprofloxacin (200mg), and metronidazole (500mg) in the ratio of 1:1:1. They were mixed in the vehicle of chlorhexidine. The mix was carried to the root canal using a carrier and was suspended into it. This mix was again activated inside the root canal using the Endoactivator. Then the cavity was packed with a small round ball of cotton followed by restoring it with temporary restoration material (Cavit G, 3M ESPE, Kamen, Germany).

**Figure 8:**
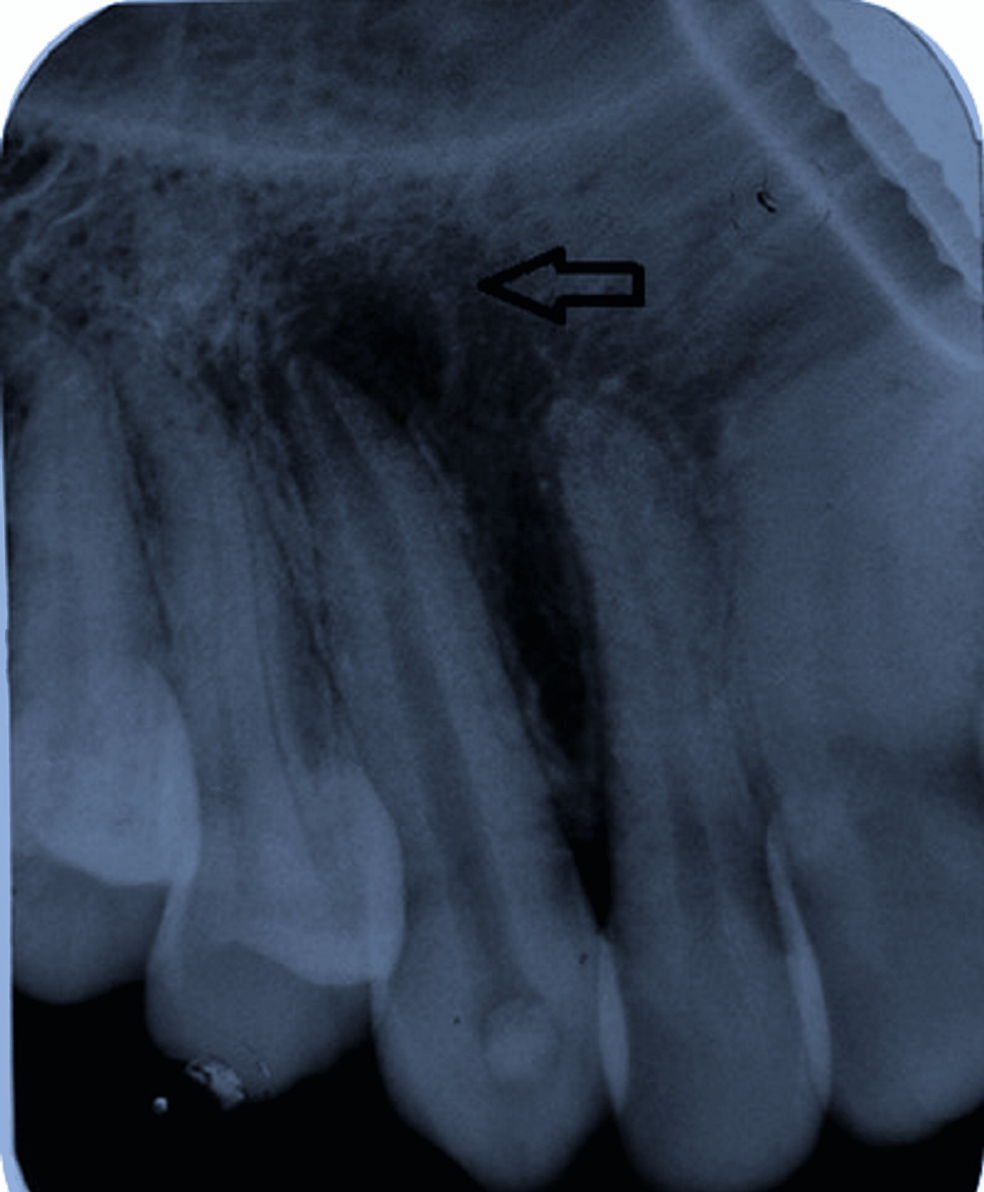
Periapical radiograph recorded at the recall period of 6 months to visualize the status of the periapical region of tooth number 13.

The patient was asked to revisit the center after two weeks each for the change of the intracanal medicament dressing of tooth number 13. Eight sittings of re-evaluation were undertaken, which included a change in the medicament dressing of tooth number 13. At each sitting, fluid discharged readily from the cavity. At the ninth sitting, this discharge stopped. Nevertheless, when a paper point (number 60, 2%) was inserted into the canal, it came out soaked in turbid fluid at the apical third. This was the sign of a weeping canal, hence obturation was contraindicated. So, another dressing of intracanal medicament was placed. The same phenomenon continued in the 10th and 11th sittings of the re-evaluation phase. Intraoperative periapical radiograph was recorded during these visits to visualize the status of the periapical region of tooth number 13 (Figure 8).

**Figure 9:**
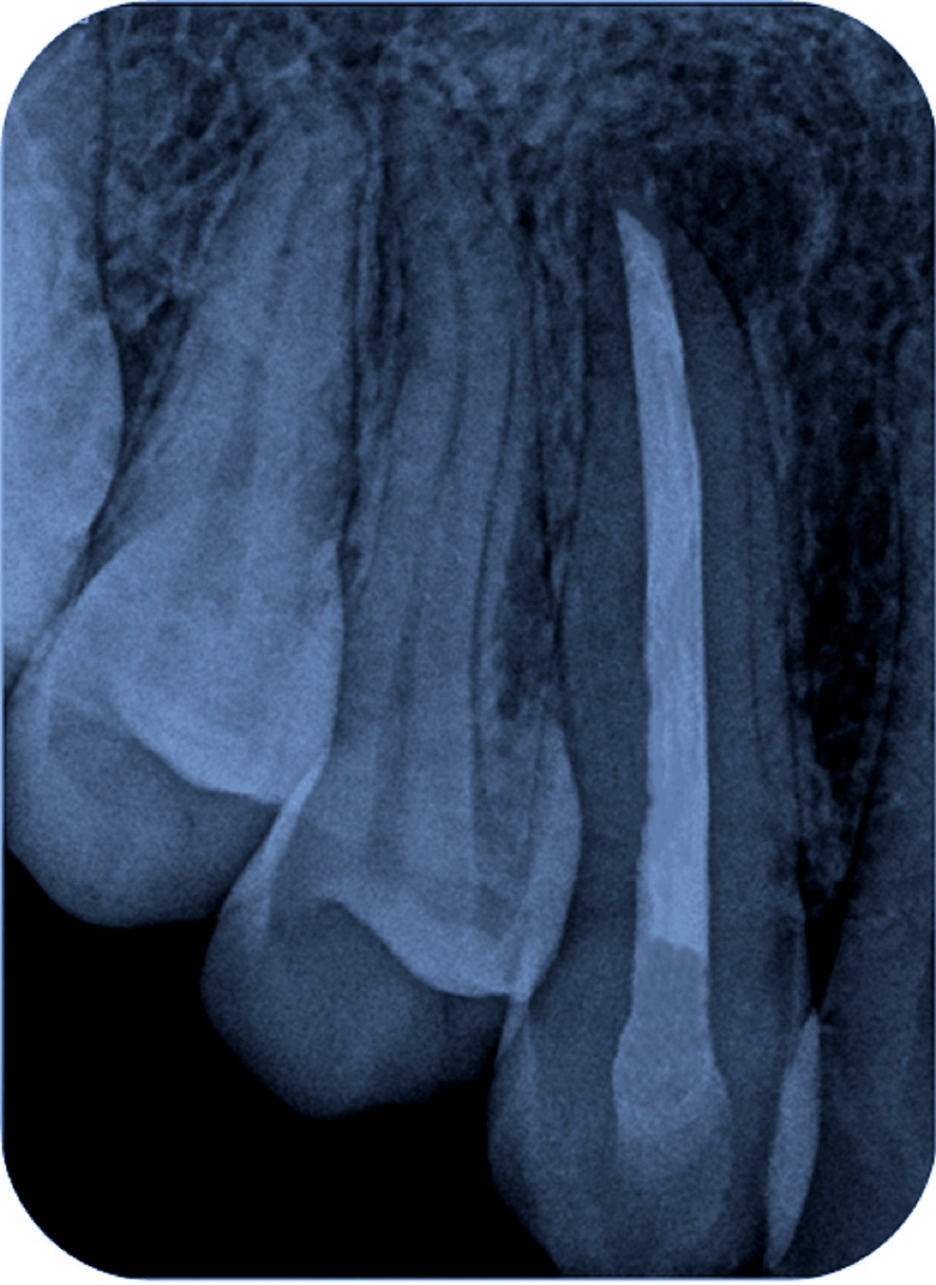
The periapical radiograph recorded at one-year follow-up. It showed a healing periapical lesion associated with tooth number 13.

At the 12th sitting, the canal was finally dry, which was verified by a paper point. However, another dressing of the same medicament was given to the canal. Thus, at the end of six months, the cold lateral compaction obturation technique was used to obturate the canal and the canal orifice tip was then sealed with GIC (GC Fuji II, Tokyo, Japan) followed by resin composite restoration (Clearfil AP-X, Kuraray, Osaka, Japan). The patient was recalled for follow-up after one, three, and six months up to one year. The patient did not show any clinically relevant signs or symptoms at the end of one year. The periapical radiograph recorded at one-year follow-up is shown in Figure 9, which showed a healing periapical lesion associated with tooth number 13.

## Discussion

Inflammatory periapical lesions of endodontic origin often have a diameter of 5 to 8 mm [11]. Lesions up to 10 mm are traditionally classified as granulomas, which have a prevalence ranging from 6% to 55%, while bigger lesions are classified as apical cysts, which have a prevalence of 15% to 32% [11]. The apical cyst is categorized as a “True” or “Bay” cyst, according to how the cavity of the cyst and the canals of the root are connected through the foramen of the apex [11, 12]. According to histopathological investigations, apical cysts and granulomas have equal frequencies of occurrence. Periapical cysts are usually caused by a microbial infection and irritation. It may grow in size due to an increase in the osmotic intensity or because of the growth of the epithelial cells [13].

Although the authors point out that surgical methods seem to be associated with increased patient discomfort, they also seem to be linked with speedy healing of the bones [14, 15]. The authors emphasize that there isn't a certain advantage of using surgical methods over nonsurgical methods and that patient preference, operator competency, cost, and technological viability should be considered when choosing a method of treatment. There is a greater discomfort in the early postoperative period after surgical invention [16].

Various intracanal medicaments such as Ledermix and Septomixine Forte are used [17]. However, studies have shown that these medicaments did not display an adequate amount of antimicrobial activity, leading to the persistence of the virulent endodontic microbiota [17, 18]. The combination of triple antibiotic paste (TAP) was initially developed by Hoshino and colleagues (1996) [19]. TAP was first created expressly to treat open apex teeth with necrotic pulp and to support the protocol for revitalization and regrowth. The treatment was very effective in disinfecting the root canal system [20]. TAP is a mixture of ciprofloxacin, metronidazole, and minocycline in the ratio of 1:1:1 to a final concentration of 0.1-1.0 mg/ml [21]. William Windley et al. noticed a notable decrease in the quantity of microorganisms in the root canal after applying antibiotic paste followed by irrigation [22]. According to this study, there was a persistence of 90% of virulent bacteria in the root canal even after the irrigation with 1.25% sodium hypochlorite. However, after two weeks of TAP application, this decreased to 30% [23]. The main aim of triple antibiotic pastes is to eliminate the wide variety of virulent endodontic microflora, which would facilitate the arrest of external inflammatory root resorption, and thus the healing of periapical lesions [24]. Additionally, it serves as a medicated sealer for protection against potential reinfection and also used as a dressing material instead of calcium hydroxide [25]. The tooth discoloration is attributed to the presence of minocycline in the intracanal medication and is mentioned as a disadvantage of the paste [26]. Thibodeau and Trope suggested Cefaclor as an alternative to minocycline in TAP [27]. Reynolds et al. reduced the discoloration by using the dentin bonding agent and composite resin before placement of TAP into the root canal [28].

Later, researchers in the field of endodontics invented the modified triple antibiotic pastes. The modified triple antibiotic paste was applied to our patient. This triple antibiotic paste is a combination of three antibiotics, i.e., metronidazole (500mg), ciprofloxacin (200mg), and clindamycin (100mg) in the ratio of 1:1:1 to a final concentration of 1mg/ml [29,30]. Each antibiotic has its mechanism of action, and together they provide broad-spectrum antimicrobial activity against a wide range of bacteria commonly associated with endodontic infections [31]. Metronidazole is an antibiotic that is particularly efficient in preventing the growth of anaerobic microbes. Ciprofloxacin is a fluoroquinolone antibiotic with wide-ranging activity against both Gram-positive and Gram-negative bacteria. Clindamycin is particularly effective against anaerobic bacteria [32]. Based on the typical healing rate of 3 mm, which takes two months, a 30 mm lesion will take 10 months to fully heal [32]. Surgical intervention needs to be considered if the lesion grows huge or heals at a lowly typical pace. The triple antibiotic paste is usually inserted inside the canal for a predetermined amount of time and then removed followed by the irrigation of the root canal. The safest concentration of the TAP is (1 mg/mL) [33]. Although larger dosages of TAP provide sufficient antibacterial effect, it might be detrimental to the stem cells of apical papilla, leading to a decrease in the potentiality of stem cells to proliferate and generate a bone matrix [34-37]. Hence, a safe dose is always recommended. In the current case scenario, the best option chosen was a nonsurgical root canal therapy. 

## Conclusions

A substantial lesion resembling a periapical cyst has been cured with nonsurgical root canal therapy, utilizing intracanal medicament, i.e., modified triple antibiotic paste and root canal obturation. The triple antibiotic paste is one of the antibiotic combinations that is effective against bacteria. This proves that nonsurgical treatment can be beneficial even for large periapical lesions. For this reason, nonsurgical treatment should always be the first treatment of choice over surgical intervention.
